# Retrospective analysis of 15 years of horse-related maxillofacial fracture data at a major German trauma center

**DOI:** 10.1007/s00068-020-01450-w

**Published:** 2020-07-22

**Authors:** Rebecca Stier, Frank Tavassol, Claudia Dupke, Maria Rüter, Philipp Jehn, Nils-Claudius Gellrich, Simon Spalthoff

**Affiliations:** 1 Department of Facial Surgery, Catholic Children’s Hospital, Wilhelmstift, Liliencronstrasse 130, 22149 Hamburg, Germany; 2 Department of Oral and Maxillofacial Surgery, Hannover Medical School, Carl-Neuberg Strasse 1, 30625 Hannover, Germany; 3 Department of Biometry and Environmental System Analysis, Faculty of Environment and Natural Resources, University of Freiburg, Freiburg, Germany

**Keywords:** Horse, Maxillofacial fractures, Protective equipment, Complications, Equestrian experience, Trauma mechanism

## Abstract

**Purpose:**

The purpose of this study was to estimate the effect of the mechanism of trauma (fall versus kick), rider demographics, equestrian experience, protective equipment, and whether or not a horse was shod on the anatomic site of a horse-related maxillofacial fracture, operating time, postoperative complication rate, and length of hospital stay.

**Methods:**

We retrospectively reviewed the medical records of patients treated for horse-related maxillofacial fractures at a single institution in Germany between January 2000 and March 2015. We used linear and logistic regression to test the above-mentioned variables for statistical correlations.

**Results:**

During the study period, we treated 138 horse-related facial fractures in 71 patients. The mean patient age was 34.5 years, and 80.3% of the injuries occurred in women. Most of the maxillofacial fractures were the result of a horse kick (71.8%) when unmounted and the majority occurred in more experienced riders (70.4%). There was a significant association of wearing of protective equipment with a shorter hospital stay and lower risk of postoperative complications.

**Conclusion:**

More education is needed in the equestrian community regarding the use of protective equipment when unmounted. Safety helmets should be redesigned to include a faceguard and be worn at all times.

## Introduction

In 2014, nearly 400,000 people in Germany required medical treatment for maxillofacial trauma [1]. Sports-related accidents are a common cause of facial injuries in Europe [2]. Equestrian sport is a popular recreational and competitive activity in European countries [3]. The reported incidence of horse-related injuries is higher than that in automobile racing, motorcycle riding, football, and skiing, and is at least as high as that in rugby. Therefore, equestrian sport is considered relatively dangerous, and the injuries sustained are often more severe than those that occur in other sporting activities. Head injuries in particular are disproportionately represented in horse-related accidents and are also cited as the major cause of mortality [4–8]. Twenty percent of all horse riders suffer serious injuries resulting in hospitalization, surgery, and/or long-term disability during their lifetime, which impose high financial burdens on the health care system. The risk of these injuries is even higher in non-professional riders [9, 10]. Nevertheless, the popularity of equestrian sport is continuing to increase with every passing year.

Previously used as a work animal, horses are now used primarily for recreational and sports activities [11]. The head of a mounted rider is approximately 3 m above the ground, which contributes to horse riding being a high-risk sport. Horse-related injuries can occur while the rider is mounted (caused by a fall from the horse) or unmounted (usually due to being crushed, trodden on, or kicked). The risk of a maxillofacial injury is particularly high in the unmounted situation for the simple reason that the face is the most vulnerable anatomic site when an equestrian is in close proximity to a horse on the ground [8, 11–14].

In recent years, there has been a steady campaign to raise awareness about safety when horse riding. Wearing a safety helmet and back protector is recommended in Germany, depending on the type of equestrian sport and skill level. In the UK, Chitnavis et al. reported a 46% reduction in horse-related injuries over a period of 20 years after safety guidelines for riders were revised. The main reason for this decrease was the reduction in number of head injuries due to wearing of a helmet [6]. Nevertheless, many riders continue to wear no protective equipment, regardless of whether they are mounted or unmounted [15]. There is debate in the literature regarding the protective effect of helmets and the risk factors for facial injuries [5, 6, 11, 14, 16, 17]. A particular criticism against riding helmets is that they are designed to withstand a force of only 80–100 J, whereas the force of a kick from a horse is around 400 J [18]. Furthermore, the face is left unprotected because normal safety helmets do not include a faceguard.

The purpose of this study was to investigate the circumstances, mechanisms, and anatomic sites of horse-related facial injuries as well as the use of protective equipment in a German urban area in an effort to inform the development of new strategies for injury prevention in equestrians. We hypothesized that most horse-related maxillofacial injuries would occur in less experienced equestrians when unmounted on the basis that they would be less trained in the handling of horses and less aware of the need to wear protective equipment. The specific aims of the study were to estimate the effects of the mechanism of trauma (fall versus kick), rider demographics, equestrian experience, wearing of protective equipment, and whether or not the horse was shod on four response variables, i.e., postoperative complication rates, operating time, length of hospital stay, and fracture site.

## Materials and methods

### Study design and sample

This retrospective observational study included patients who had undergone surgery for horse-related facial fractures in the Department of Craniomaxillofacial Surgery at Hannover Medical School between January 2000 and March 2015 and for whom complete data were available. The patients were identified by searching the hospital’s medical database for ICD codes pertaining to facial fractures (Table 1) and filtering for horse-related trauma. Patients with soft tissue injuries only, patients who were crushed by horses, and those who were treated conservatively were excluded. The study protocol was approved by the regional Ethical Review Board of Hannover Medical School and was conducted in accordance with the tenets of the Declaration of Helsinki. Informed consent was not required for this study because all patients who receive treatment at our institution consent to use of their clinical data for research purposes as a part of their contract with the hospital.

**Table 1:** ICD codes used to identify horse-related maxillofacial fractures during the study period

ICD	Diagnosis
S02.0	Skull roof fractures
S02.1	Skull base fractures
S02.2	Nose fractures
S02.3	Orbital fractures
S02.4	Fractures of the maxilla and zygoma
S02.5	Tooth fracture
S02.6	Mandibular fractures
S02.7	Multiple fractures of the skull and facial bones
S02.8	Fractures of other skull and facial bones
S02.9	Fractures of the skull and facial bones, parts not defined

### Study variables

The data set contained information on the mechanism of injury (fall or hoof kick, whether or not the horse was shod), fracture site (mandible, maxilla, zygoma, orbit, and/or nose), patient demographics (age and sex), medical details on associated injuries (brain, abdominal, thoracic, limb, and/or multiple trauma), self-reported experience of the rider (novice, advanced, or professional), and use of protective equipment (helmet and/or vest).

### Statistical analysis

A descriptive analysis was performed for the demographic data. We used linear and logistic regression to test the significance of multiple dependent variables (Table 2) on the four response variables, namely postoperative complication rate, operating time, length of hospital stay, and fracture site. We tested for collinearity between variables used in the same model and excluded those that were problematic. The postoperative complication rates were fitted to a generalized linear model with a binomial link and included explanatory factors (type of trauma, rider age and sex, level of experience, whether or not protective equipment was worn, whether or not the horse was shod, and other injuries). The effects of the other above-mentioned variables on the logarithmized operating time and logarithmized length of hospital stay were tested in a linear regression model. Interactions between trauma and whether or not the horse was shod and between trauma and whether or not protective equipment was worn at the time of injury were tested for significance in all the models. The outcome at the fracture site was tested against several potential explanatory factors using a generalized linear model with a multinomial link and a binomial link after aggregating sites for the midface and mandible. Models that included more than four predictors were selected by stepwise regression analysis using a backward selection procedure based on the Akaike information criterion value. All statistical analyses were performed using R statistical software (R Core Team [2018]. R: A language and environment for statistical computing. R Foundation for Statistical Computing, Vienna, Austria) on a Linux machine. A *p* value ≤ 0.05 was considered statistically significant.

**Table 2:** Statistical linear and logistic regression models used in this study

Outcome (response) variables	Predictor variables	Interactions
Postoperative complication rate	Type of trauma Rider age and sex Rider experience Wearing of protective equipment Whether or not the horse was shod	Type of trauma and horse shoeing status, type of trauma and wearing of safety equipment
Operating time	Type of trauma Rider age and sex Rider experience Wearing of protective equipment Whether or not the horse was shod	
Length of stay	Type of trauma Rider age and sex Rider experience Wearing of protective equipment Whether or not the horse was shod	
Fracture site	Type of trauma Rider age and sex Rider experience Wearing of protective equipment Whether or not the horse was shod	

## Results

Seventy-one patients had undergone surgical treatment for 138 horse-related maxillofacial fractures during the 15-year study period. Fifty-seven (80.3%) of the 71 patients were female and 14 (19.7%) were male (giving a female–male ratio of 4.1:1). The mean patient age was 34.5 years (range, 7–78 years; standard deviation, ± 16.6). During the study period, we treated approximately 200 patients with maxillofacial fractures per anno, resulting in a prevalence of horse-related facial fractures of approximately 2.37%. More than 50% of the injuries occurred in the final 6 years of the study. Table 3 shows the distribution of fracture type according to patient demographics (age and sex), timing of the accident (season, day of the week, and time of day), associated injuries (brain, abdominal, thoracic, limb, and/or multiple trauma), self-reported equestrian experience of the rider (novice, advanced, or professional), and use of protective equipment (helmet and/or vest).

**Table 3:** Associations between the fracture site and other study variables

	Total patients (*n* = 71)	Total fractures (*n* = 138)	Fractured orbit (*n* = 37)	Fractured zygoma (*n* = 27)	Fractured nose (*n* = 23)	Fractured maxilla (*n* = 31)	Fractured mandible (*n* = 20)
Age, years							
Child < 13	6	10	3	2	1	2	2
Adolescent 13–17	5	10	3	3	3	1	0
Adult 18–40	35	64	13	11	10	16	14
Adult 41–65	23	50	17	10	9	11	3
Adult > 66	2	4	1	1	0	1	1
Sex							
Male	14	27	10	5	4	5	3
Female	57	111	27	22	19	26	17
Protective equipment							
Helmet^a^	17	31	9	8	4	6	4
Helmet and vest	2	2	2	0	0	0	0
None	36	73	17	12	14	18	12
Associated injuries							
Brain	28	63	20	16	10	13	4
Cervical spine	2	4	0	0	0	2	2
Thoracic	2	3	2	0	0	1	0
Extremity	2	6	2	2	0	1	1
Polytrauma	3	5	1	0	1	1	2
None	36	57	12	9	12	13	11
Skill level							
Novice^a^	9	12	4	2	1	4	1
Advanced	30	61	17	14	9	14	7
Professional	20	38	8	6	8	8	8

^a^Numbers may not always add up because of missing information

Most of the injuries occurred in patients who described themselves as experienced or professional riders (70.4%; unknown in 12). Protective clothing was worn in 34.5% of cases. Helmets and vests were worn by 67% of beginners but by only 44% of advanced riders and 33% of professional riders. Consequently, experienced riders used protective equipment significantly less often (beginners vs. advanced riders, *p* = 0.05; beginners vs. professional riders, *p* = 0.03).

A kick was the most common cause of fracture at all facial sites. Approximately two-thirds (67%) of fractures caused by kicks were localized to the midface, with the remainder being localized to the mandible. The majority (85%) of fractures resulting from falls affected the midface. No factors were significantly associated with the site of fracture.

**Fig. 1:**
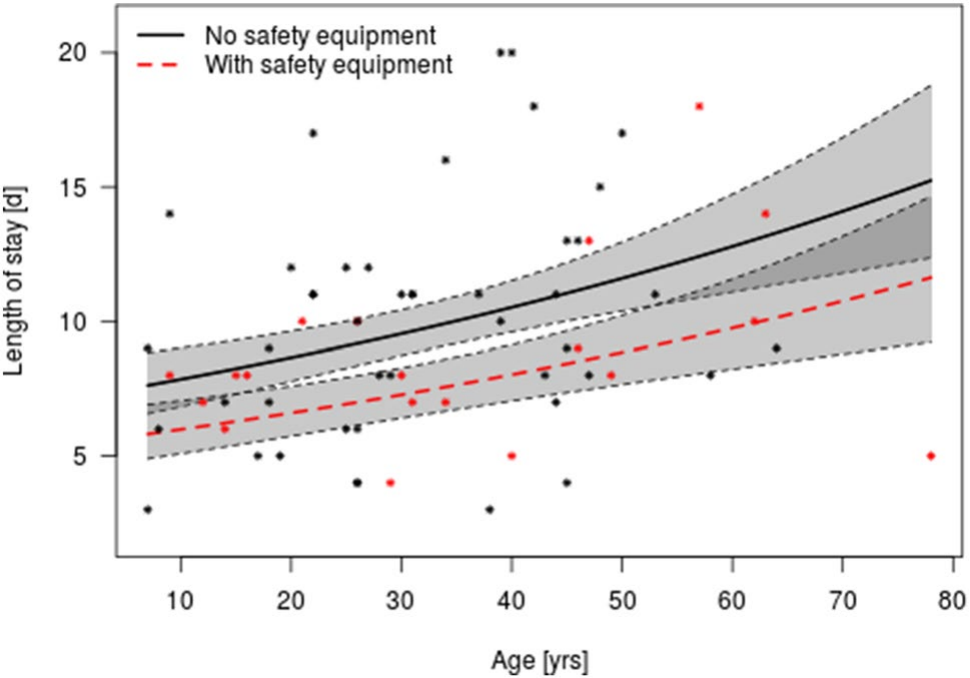
Effect of age and use of protective equipment on the length of hospital stay after a horse-related maxillofacial fracture. The lines refer to the predictions of a linear regression where length of hospital stay was logarithmized (here the prediction is re-transformed) based on a data set of 55 patients (16 patients gave no information on their protective equipment), according to whether or not they were wearing protective equipment at the time of the accident. The black solid line indicates the patients who were not wearing protective equipment and the red line indicates those who were wearing protective equipment. The shaded gray areas represent the confidence interval (with standard error of the mean)

In 7 of the 51 cases caused by a hoof kick, the horse involved was shod; in 31 cases, the horse was not shod (it was unknown if the horse was shod or not in 13 cases). There was no statistically significant difference in operating time (*p* = 0.39) or postoperative length of hospital stay (*p* = 0.73) according to whether or not the horse was shod. However, when confounding factors such as rider age, sex, experience, and other injuries were accounted for when testing the effects on operating time and length of hospital stay, only age (*p* = 0.03) and whether or not protective equipment was worn (*p* = 0.08) had marked effects on length of hospital stay (Fig. 1). The older the patient, the longer the hospital stay. Wearing of protective equipment at the time of the accident decreased the average length of hospital stay by a factor of 0.75.

**Fig. 2:**
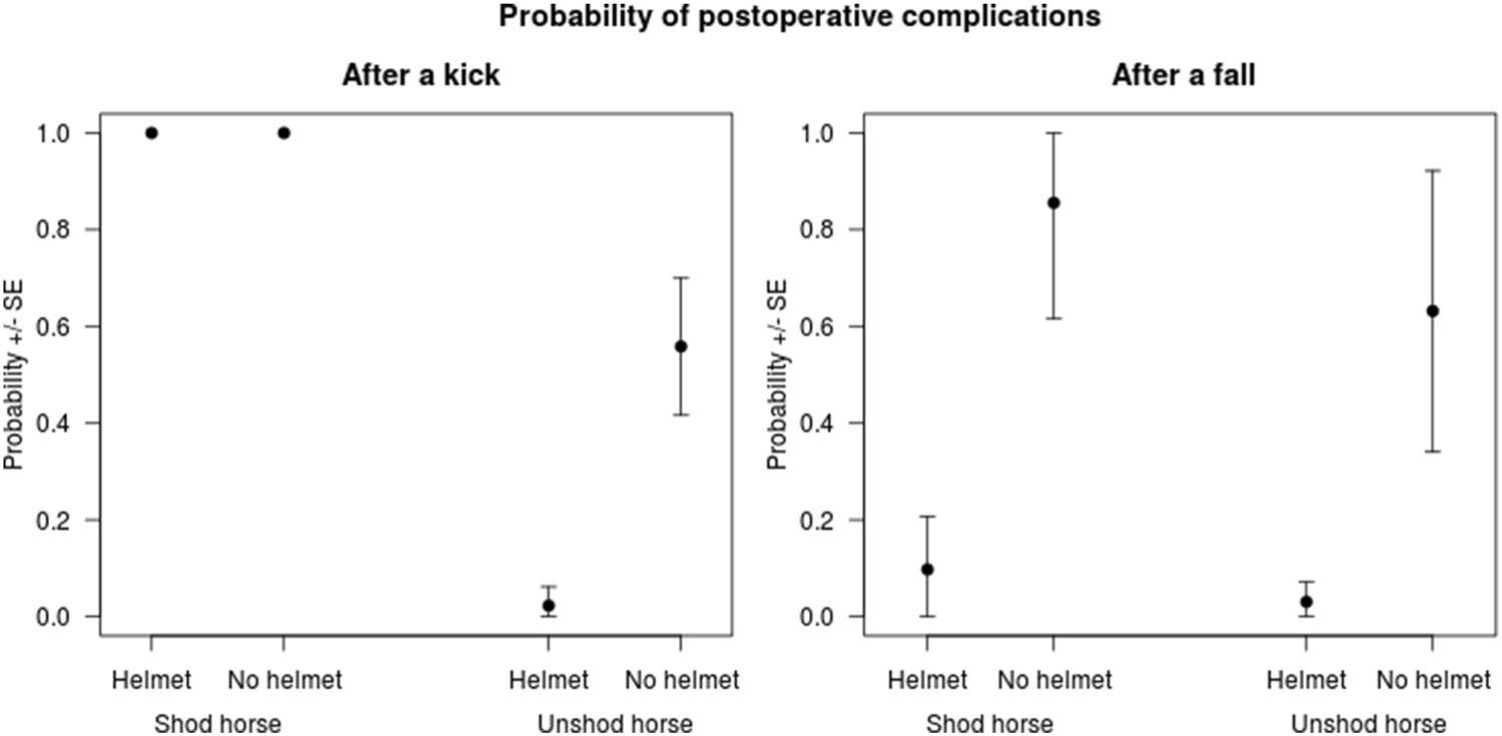
Probability of postoperative complications according to type of trauma (left, kick; right, fall), whether the horse was shod or not, and if a helmet was worn. Probabilities are derived from a logistic regression model based on all 71 events. Error bars show the associated standard error of the mean

All riders kicked by a shod horse (*n* = 7) developed complications after surgery. In all other cases, the likelihood of postoperative complications was reduced if a helmet was worn; 29 (81%) of 36 patients who were not wearing a helmet at the time of the accident developed complications; whereas, only 5 (29%) of 17 who were wearing a helmet developed complications (Fig. 2). Other factors tested, including sex, age, level of experience, and whether or not other injuries were present, had no significant effects on the complication rate either as a single explanatory variable or in combination with other variables.

## Discussion

In this study, we investigated the circumstances, mechanisms, and sites of horse-related facial injuries, as well as the use of protective equipment in a German urban area. Our hypothesis was that most of the maxillofacial injuries occurring in equestrians would be sustained by less experienced riders when unmounted. This theory was based on their relative lack of experience in handling horses and their presumed lack of awareness of the need to wear protective equipment. The specific aims were to estimate the effects of the mechanism of trauma (fall versus kick), rider demographics, equestrian experience, wearing of protective equipment, and whether or not the horse was shod on four response variables, namely fracture site, operating time, postoperative complication rate, and length of hospital stay.

The finding that most horse-related maxillofacial fractures occurred when the rider was not mounted or wearing protective equipment partially confirmed our hypothesis. However, the finding that the majority of injuries were sustained by professional riders rather than novices was unexpected. Moreover, experienced riders were less likely to have been wearing a safety helmet or vest at the time of the injury.

Maxillofacial fractures are usually the result of interpersonal violence, motor vehicle accidents, sports, or falls and are mainly sustained by young men [2, 16]. Horse riding is unique in this regard, because it is the only sport in which women are injured more often than men [5, 6, 10, 13]. Equestrians who sustain horse-related injuries are typically young women aged around 30 years who ride for recreation and know their mounts well [14, 17]. Our finding that 80.3% of the injuries occurred in women is consistent with most published reports [5, 6, 10, 13, 15]. More than 50% of the injured equestrians in our study were aged 18–40 (mean 34.5) years, which is again similar to the data in the literature [16]. In line with the increasing popularity of equestrian sport and the greater number of riders [3], more than half of the documented injuries occurred in the final 6 years of our study.

Facial fractures have been reported to account for 19s–54% of all horse-related injuries [17, 19]. These injuries are associated with lengthy hospitalization, need for surgery, long-term disability, and a high financial burden to the health care system [3, 9, 10]. We investigated 138 facial fractures in 71 patients and found that in most cases, the fracture occurred in the midface, particularly the orbit. This finding is similar to that of a previous study, which reported a midfacial fracture rate of 58% [20]. Similar results were reported by Lee and Steenberg and by Antoun et al. [14, 16]. Statistically, no factors were significantly associated with the site of fracture.

The high incidence of horse-related facial fractures can be explained by the mechanism of trauma. In our study, most (71.8%) of the injuries were caused by hoof kicks. A kick from a horse can transfer a force of 10,000 N to the human body, and the face is the most common site of injury [11, 14]. Meredith et al. investigated maxillofacial fractures in unmounted equestrians and found that a hoof kick was the mechanism in 69% of cases [12]. Similar results were found in the study by Islam et al., in which 71% of all injuries were caused by hoof kicks [20]. Weber et al. also found that horse kicks were the leading cause of midfacial fractures [21], the most common of which were orbital fractures. In a study by Eckert et al., 66.7% of riders with facial injuries caused by hoof kicks sustained fractures of the orbit, midface, and/or mandible [22].

Surprisingly, there was no statistically significant effect on the operation time concerning all the analyzed dependent variables. We had expected increased operation times when the horses were shod due to more severe facial trauma caused by increased force transmission. However, this assumption was not statistically proven.

Nevertheless, there was some influence on the overall severity of horse-related trauma. Injuries resulting from kicks by shod horses were always associated with postoperative complications (e.g., an infection, nerve lesion, or blindness), reflecting more severe, blunt-force trauma inflicted by horses wearing metal shoes. Wearing of safety helmets had no influence on the risk of postoperative complications subsequent to kicks by shod horses but was associated with decreased risk of complications subsequent to kicks from unshod horses. Our findings support the recommendation that protective equipment should be worn at all times whether the rider is mounted or unmounted.

Interestingly, the length of postoperative hospital stay was longer not only for older patients, which is already a widely known fact, but also for patients who were not wearing protective equipment at the time of injury, which suggests that protective equipment may protect against more severe injury patterns.

Despite horse riding being a high-risk sport, protective equipment is rarely used by equestrians, regardless of whether they are mounted or unmounted. In our study, only 34.5% of the patients were wearing helmets and/or back protectors at the time of injury. Other studies have reported helmet compliance rates of 6–61% [9, 13, 20]. Since the compliance rate does not seem to be time dependent, the increase in horse-related injuries over recent years possibly reflects the increasing popularity of equestrian sport. The literature consistently shows that safety helmets have a protective effect against brain injuries [6, 8, 22]. In the UK, Chitnavis et al. reported a 46% reduction in horse-related injuries over a period of 20 years because of the revised safety guidelines for riders [6]. Only one study has investigated the potential benefit of wearing a helmet to protect the face, and its findings were negative [17]. Even with a helmet, the face is still vulnerable to a horse-related injury, especially a hoof kick. Therefore, in our opinion, a helmet cannot protect against facial injuries unless it is fitted with a faceguard. The common reasons cited by equestrians for not wearing a helmet are that they are unnecessary (43.8%) or uncomfortable (29.9%) [23]. In our study, we did not find a consistent relationship between length of hospital stay and wearing protective equipment in patients who had been kicked by shod horses, but the hospital stay was shorter in all other patients who had been wearing helmets. This finding suggests that helmets offer some protection against kicks by unshod horses, which are known to cause less severe injuries than kicks by horses wearing metal shoes.

It is estimated that 20% of all equestrians have an injury at some point in their riding career, with the risk being highest in novices [9]. However, in our study, 70.4% of the injuries were sustained by riders who self-reported as advanced or professional and only 12.7% by novices, which is contrary to our hypothesis. Individuals who ride for a living have more contact with horses, including horses that are excitable and not necessarily well handled. Hence, their risk of injury may be higher. Furthermore, many adult horse riders are women who started riding for recreation at young ages and have accumulated many years of experience around horses. It is possible that their risk awareness decreases over time due to constant contact with horses, especially when unmounted. It has been reported that 18–64% of horse-related injuries could be avoided by more vigilance and experience on the part of the rider, better training of horses, and routine use of protective equipment [24]. Half of the injured equestrians in our study had additional injuries. Islam et al. similarly reported that about 33% of their patients had other injuries [20]. This is important information for clinicians, and hence, we recommend a multidisciplinary examination for all patients with horse-related trauma.

We believe that more education and preventative measures are needed to prevent horse-related injuries in the equestrian community. All equestrians need to be knowledgeable about horsemanship and should have good handling skills, especially when unmounted, and know the situations in which an injury is more likely to occur. They must recognize that experience does not protect against injury. Ongoing training for novices combined with close supervision by more experienced riders, and regular reminders for industry professionals concerning safety measures are needed. Helmets need to incorporate a face shield and should be worn at all times.

This study had some limitations, which stem mainly from its retrospective design, relatively small sample size, and the collection of data from only one urban center. First, we cannot be certain whether or not all patients’ experience in terms of riding and handling horses was accurately recorded. Second, we cannot be certain that all the patients in the study who were injured whilst unmounted were actually horse riders. The number of injuries that occurred in non-riding individuals was unknown. Third, the study was inevitably uncontrolled, so we were unable to assess the actual protective effect of a safety helmet. Worthwhile avenues of research in the future could include establishing whether or not the passing of legislation to ensure more safety training and use of modified protective equipment at all times would alter the frequency and severity of horse-related maxillofacial fractures.

In summary, horse-related maxillofacial fractures are often the result of horse kicks and are more likely to occur in experienced equestrians not wearing protective helmets while handling horses on the ground. The hospitalization time was longer not only in older patients, as expected but also in those who were not wearing a protective helmet at the time of injury. These findings underscore the importance of more education and reminders in the equestrian community regarding the use of protective equipment and safety procedures, especially when unmounted.

## References

[1] Federal Bureau for Statistics, Germany. 2016.

[2] Boffano P, Roccia F, Zavattero E, Dediol E, Uglešić V, Kovačič Z (2015). European Maxillofacial Trauma (EURMAT) project: a multicentre and prospective study. J Craniomaxillofac Surg.

[3] Schröter C, Schulte-Sutum A, Busch M, Winkelmann M, Macke C, Zeckey C (2017). Cervical spine injury in equestrian sports. Der Unfallchirurg..

[4] Ball CG, Ball JE, Kirkpatrick AW, Mulloy RH (2007). Equestrian injuries: incidence, injury patterns, and risk factors for 10 years of major traumatic injuries. Am J Surg.

[5] Hasler RM, Gyssler L, Benneker L, Martinolli L, Schötzau A, Zimmermann H (2011). Protective and risk factors in amateur equestrians and description of injury patterns: a retrospective data analysis and a case-control survey. J Trauma Manag Outcomes..

[6] Chitnavis JP, Gibbons CL, Hirigoyen M, Parry JL, Simppson ARHW (1996). Accidents with horses: what has changed in 20 years?. Injury.

[7] Paix BR (1999). Rider injury rates and emergency medical services at equestrian events. Br J Sports Med.

[8] Whitlock MR (1999). Injuries to riders in the cross country phase of eventing: the importance of protective equipment. Br J Sports Med.

[9] Mayberry JC, Pearson TE, Wiger KJ, Diggs BS, Mullins RJ (2007). Equestrian injury prevention efforts need more attention to novice riders. J Trauma.

[10] Altgärde J, Redéen S, Hilding N, Drott P (2014). Horse-related trauma in children and adults during a two year period. Scand J Trauma Resusc Emerg Med..

[11] Exadaktylos AK, Eggli S, Inden P, Zimmermann H (2002). Hoof kick injuries in unmounted equestrians. Improving accident analysis and prevention by introducing an accident and emergency based relational database. Emerg Med J..

[12] Meredith L, Antoun JS (2011). Horse-related facial injuries: the perceptions and experiences of riding school. Inj Prev..

[13] Carmichael SP, Davenport DL, Kearney PA, Bernard AC (2014). On and off the horse: mechanisms and patterns of injury in mounted and unmounted equestrians. Injury.

[14] Antoun JS, Steenberg LJ, Lee KH (2011). Maxillofacial fractures sustained by unmounted equestrians. Br J Oral Maxillofac Surg.

[15] Davidson SB, Blostein PA, Schrotenboer A, Sloffer CA, VanderBerg SL (2015). Ten years of equine-related injuries: severity and implications for emergency physicians. J Emerg Med.

[16] Lee KH, Steenberg LJ (2008). Equine-related facial fractures. Int J Oral Maxillofac Surg.

[17] Ueeck BA, Dierks EJ, Homer LD, Potter B (2004). Patterns of maxillofacial injuries related to interaction with horses. J Oral Maxillofac Surg.

[18] Fleming PR, Crompton JL, Simpson DA (2001). Neuro-ophthalmological sequelae of horse-related accidents. Clin Exp Ophthalmol..

[19] Sorli JM (2000). Equestrian injuries: a five year review of hospital admissions in British Columbia, Canada. Inj Prev..

[20] Islam S, Gupta B, Taylor CJ, Chow J, Hoffmann GR (2014). Equine-associated maxillofacial injuries: retrospective 5-year analysis. Br J Oral Maxillofac Surg.

[21] Weber CD, Nguyen AR, Lefering R, Hofman M, Hildebrand F, Pape HS (2017). Blunt injuries related to equestrian sports: results from an international prospective trauma database analysis. Int Orthop.

[22] Eckert V, Lockemann U, Puschel K, Meenen N, Christian H (2011). Equestrian injuries caused by horse kicks: first results of a prospective multicenter study. Clin J Sport Med.

[23] Nelson DE, Rivara FP, Condie C (1994). Helmets and horseback riders. Am J Prev Med.

[24] Ball JE, Ball CG, Mulloy RH, Datta I, Kirkpatrick AW (2009). Ten years of major equestrian injury: are we addressing functional outcomes?. J Trauma Manag Outcomes..

